# Fed-Batch Production of *Saccharomyces cerevisiae* L-Asparaginase II by Recombinant *Pichia pastoris MUT*^*s*^ Strain

**DOI:** 10.3389/fbioe.2019.00016

**Published:** 2019-02-08

**Authors:** David Rodrigues, Omar Pillaca-Pullo, Karin Torres-Obreque, Juan Flores-Santos, Ignacio Sánchez-Moguel, Marcela V. Pimenta, Tajindar Basi, Attilio Converti, André M. Lopes, Gisele Monteiro, Luís P. Fonseca, Adalberto Jr. Pessoa

**Affiliations:** ^1^Bioengineering Department of Instituto Superior Técnico, Institute of Bioengineering and Biosciences, Universidade de Lisboa, Lisbon, Portugal; ^2^Department of Pharmaceutical-Biochemical Technology, School of Pharmaceutical Sciences, University of São Paulo, São Paulo, Brazil; ^3^Department of Pharmacy, King's College London, London, United Kingdom; ^4^Department of Civil, Chemical and Environmental Engineering, Genova, Italy; ^5^Faculty of Pharmaceutical Sciences, University of Campinas, Campinas, Brazil

**Keywords:** L-Asparaginase, *Pichia pastoris*, defined medium, fed-batch fermentation, high cell density culture, heterologous protein production

## Abstract

L-Asparaginase (ASNase) is used in the treatment of acute lymphoblastic leukemia, being produced and commercialized only from bacterial sources. Alternative *Saccharomyces cerevisiae* ASNase II coded by the *ASP3* gene was biosynthesized by recombinant *Pichia pastoris MUT*^*s*^ under the control of the *AOX1* promoter, using different cultivation strategies. In particular, we applied multistage fed-batch cultivation divided in four distinct phases to produce ASNase II and determine the fermentation parameters, namely specific growth rate, biomass yield, and enzyme activity. Cultivation of recombinant *P. pastoris* under favorable conditions in a modified defined medium ensured a dry biomass concentration of 31 g_dcw_.L^−1^ during glycerol batch phase, corresponding to a biomass yield of 0.77 g_dcw._g${}_{\text{glycerol}}^{-1}$ and a specific growth rate of 0.21 h^−1^. After 12 h of glycerol feeding under limiting conditions, cell concentration achieved 65 g_dcw._L^−1^ while ethanol concentration was very low. During the phase of methanol induction, biomass concentration achieved 91 g_dcw._L^−1^, periplasmic specific enzyme activity 37.1 U.g${}_{\text{dcw}}^{-1}$, volumetric enzyme activity 3,315 U.L^−1^, overall enzyme volumetric productivity 31 U.L^−1^.h^−1^, while the specific growth rate fell to 0.039 h^−1^. Our results showed that the best strategy employed for the ASNase II production was using glycerol fed-batch phase with pseudo exponential feeding plus induction with continuous methanol feeding.

## Introduction

L-Asparaginase or ASNase (EC 3.5.1.1, L-asparagine amidohydrolase) is the enzyme that catalyzes the conversion of L-asparagine to aspartic acid and ammonia. It is used as a fundamental drug in protocols for the treatment of acute lymphoblastic leukemia (ALL). Up to now, only bacterial enzymes either modified (PEGylated) from *Escherichia coli* or unmodified from *E. coli* and *Erwinia chrysanthemi* have been approved for ALL treatment (Ali et al., 2016; Lopes et al., 2017; Santos et al., 2017). Even with all the success achieved with bacterial ASNases, there are still adverse effects (Dunlop et al., 1978, 1980; Panosyan et al., 2004; Liu et al., 2016); therefore, there is a demand for new serologically different enzymes with improved characteristics such as less immunogenicity with similar or improved therapeutic effects (Narta et al., 2007). For this purpose, *Saccharomyces cerevisiae* ASNase II significantly differs in several aspects from the bacterial enzymes, showing higher stability, optimum pH close to physiologic human conditions and lower allergenic potential (Jones, 1977; Dunlop et al., 1978, 1980; Ferrara et al., 2006). Although *S. cerevisiae* is able to add post-translational modification to proteins such as glycosylation, it causes hyperglycosylation that increases immunogenicity (Looser et al., 2015) and has lower secretory capacity compared to *Pichia pastoris* (Zhang et al., 2000a).

*Pichia pastoris* has been widely used for the expression of more than 1,000 heterologous proteins (Itzel et al., 2016). The success of this yeast as an expression system is related to its ability to reach high cell densities (Pichia Fermentation Process Guidelines, 2002) on simple, inexpensive and chemically-defined media as well as the use of simple techniques for its genetic manipulation (Cereghino and Cregg, 2000). Among the various advantages that make *P. pastoris* so attracting in heterologous protein production are protein processing and post-translational modification that allow for eukaryotic protein proper folding and activity (Itzel et al., 2016; Sun et al., 2016). In addition, this yeast has a well-developed secretory system, prefers respiratory growth, and produces little ethanol (Cereghino and Cregg, 2000).

At the transcriptional level, the carbon source plays an important role in regulating enzyme synthesis (Brierley et al., 1990), because catabolite repression is exerted by many compounds, especially glucose and ethanol (Meagher and Inan, 2001). Cultivation of recombinant *P. pastoris* expressing a product under the control of the highly regulated Alcohol Oxidase I (*AOX1*) promoter in high cell density cultures is often carried out according to a multi-stage fermentation protocol (Zhang et al., 2006). Typically, this process is divided into two major stages: growth and induction/production (Brierley et al., 1990; Chiruvolu et al., 1998; Cereghino and Cregg, 2000; Pichia Fermentation Process Guidelines, 2002). In the growth stage, a large cell mass is obtained using glycerol as a carbon source, because *P. pastoris* grows significantly faster in this substrate than in methanol (Looser et al., 2015). This stage is divided in two phases: glycerol batch phase (GBP) and glycerol fed-batch phase (GFP). The recombinant protein induction and production take place during a methanol fed-batch phase (MFP) (Looser et al., 2015), which is essential to prevent either overfeeding or underfeeding of substrate to the culture medium (Dietzsch et al., 2011; Looser et al., 2015).

Based on this background, we investigated in this study the production of ASNase by a methylotrophic recombinant *P. pastoris* strain carrying the *ASP3* gene that encodes for *S. cerevisiae* ASNase II. Different glycerol and methanol feeding strategies, which are known to play a key role on heterologous protein production by recombinant microorganisms, were investigated. The main aim of this study was to develop a protocol to achieve high cell density cultures and determine the kinetic fermentation parameters for further development of this process.

## Materials and Methods

### Microorganism, Gene Cloning, and Protein Expression

The *ASP3* gene lacking the periplasmic signaling sequence (amino acids 1–25) was first cloned into the *pET22b* vector (Novagen, San Diego, CA, USA) for C-terminal histidine tail insertion. This construct was used as template to amplify the insert *ASP3*_26-362 + his using the following primers: forward AGCGGGCCTAGGGAAGAGAAGAATTCTTC by inserting the restriction site *Avr II* and reverse AAGGAAAAAAGCGGCCGCGGATCTCAGTG by inserting the *NotI* site. This insert was then digested with the *Avr II* and *Not I* enzymes and cloned into the *pPIC9K* vector (Invitrogen, Carlsbad, CA, USA) at these restriction sites. The constructs were confirmed by sequencing. The correct clone was linearized with the *Sal I* restriction enzyme and transformed into the *P. pastoris* strain KM71 (arg4 his4 *AOX1*::ARG4) (Invitrogen) by electroporation. Transformants were selected for geneticin resistance (G418 at concentrations increasing from 0.25 to 4 mg/mL). The clone with resistance to 4 mg/mL had its genomic DNA extracted with PureLink® Genomic DNA Mini kit (Life Technologies, Carlsbad, CA, USA). Integration of the *pPIC9K* + *ASP3*_26-362 + his construct was confirmed by PCR using the primers flanking the insertion site in the genome at the *AOX* locus (forward GACTGGTTCCAATTGACAAGC and reverse GCAAATGGCATTCTGACATCC). For more details about the steps of plasmid construction, gene sequence, and vector diagram, please see the Supplementary Information section.

### *Pichia pastoris* Cell Line Preservation and Reactivation

To produce a working cell bank, the yeast strain was reactivated in yeast peptone dextrose (YPD) agar under incubation for 24 h at 30°C. The activated cells were transferred to a 250-mL Erlenmeyer flask containing 100 mL of buffered glycerol complex medium (BMGY) [composition: 10 g.L^−1^ yeast extract, 20 g.L^−1^ peptone, 100 mM potassium phosphate, pH 6.0, 3.4 g.L^−1^ yeast nitrogen base medium, 10 g.L^−1^ (NH_4_)_2_SO_4_, 4 mg.L^−1^ biotin, and 10 g.L^−1^ glycerol]. The culture was incubated in an orbital shaker (New Brunswick Scientific Excella® E24, Edison, NJ, USA) at 30°C and 250 rpm until an optical density >30 was reached. Cells were then stored at −80°C in BMGY enriched with 20% (v/v) glycerol in 1.5-mL microtubes. To prepare the pre-inoculum, cells were thawed and inoculated in a 250-mL Erlenmeyer flask containing 50 mL of BMGY medium under the same conditions as above.

### Kinetic Parameters of Growth and Yield of Biomass

Biomass specific growth rate (μ) was estimated from the experimental data of biomass concentration by the equation (Sabo et al., 2019):

(1) $$\mu =\frac{\text{1}}{\left({\text{T}}_{\text{f-}}{\text{T}}_{\text{i}}\right)}\ln\frac{{\text{X}}_{\text{f}}}{{\text{X}}_{\text{i}}}$$

where *X*_*f*_ and *X*_*i*_ are the final and initial biomass concentrations, and *T*_*f*_ and *T*_*i*_ the mean final and initial times, respectively.

The yield of biomass (Y_x/s_) on consumed substrate was defined as:

(2) $${\text{Y}}_{\frac{\text{x}}{\text{s}}}=\frac{\left({\text{X}}_{\text{f}}{\text{-X}}_{\text{i}}\right)}{\left({\text{S}}_{\text{f}}{\text{-S}}_{\text{i}}\right)}$$

where S_f_ and S_i_ are the final and initial glycerol concentrations, respectively.

### Cultures of *Pichia pastoris* in Shake Flasks

Cultures were carried out using a previously-prepared pre-inoculum where the frozen suspension was reactivated. After inoculation with a dry cell weight (dcw) concentration of about 1 g_dcw_.L^−1^, 250-mL Erlenmeyer flasks containing 50 mL of BMGY or of defined medium [modified basal salt medium (BSMm) or modified salt fermentation medium (SFMm)] were incubated as above until glycerol depletion. A 50-mL aliquot of fermented broth was centrifuged at 3,320 *g* and 4°C for 10 min and resuspended in 250-mL Erlenmeyer flasks containing 50 mL of buffered methanol complex medium (BMMY) having the same composition as BMGY except for 30 mL.L^−1^ methanol instead of 10 g.L^−1^ glycerol. The ASNase II induction phase was carried out at 20°C and 250 rpm on the same medium as that used for the growth phase, by two pulse additions of 30 mL.L^−1^ of pure methanol at the start and after 24 h. The induction phase was ended after 48 h.

BSMm contained 26.7 mL.L^−1^ of 85% (v/v) H_3_PO_4_, 0.93 g.L^−1^ CaSO_4_, 18.2 g.L^−1^ K_2_SO_4_, 14.9 g.L^−1^ MgSO${}_{4}^{.}$7H_2_O, 4.13 g.L^−1^ KOH, 13–20 g.L^−1^ (NH_4_)_2_SO_4_, and 4.35 mL.L^−1^ *Pichia* trace metal solution (PTM) (composition: 6.0 g.L^−1^ CuSO${}_{4}^{.}$5H_2_O, 0.088 g.L^−1^ KI, 3.0 g.L^−1^ MnSO${}_{4}^{.}$H_2_O, 0.2 g.L^−1^ Na_2_MoO${}_{4}^{.}$2H_2_O, 0.02 g.L^−1^ H_3_BO_3_, 0.5 g.L^−1^ CoCl_2_, 20.0 g.L^−1^ ZnCl_2_, 65.0 g.L^−1^ FeSO${}_{4}^{.}$7H_2_O, 0.2 g.L^−1^ biotin, 5.0 mL.L^−1^ concentrated H_2_SO_4_, and pH adjusted to 5.0 with NaOH), while SFMm contained 12 g.L^−1^ KH_2_PO_4_, 4.7 g.L^−1^ MgSO${}_{4}^{.}$7H_2_O, 0.36 g.L^−1^ CaCl${}_{2}^{.}$2H_2_O, 13–20 g.L^−1^ (NH_4_)_2_SO_4_, and 4.35 ml.L^−1^ PTM (pH adjusted to 5.0 with NaOH).

### 3^3−1^ Fractional Factorial Design for the Induction Phase in Shake Flasks

The induction phase was investigated through experiments carried out according to a Box–Behnken design (Box and Behnken, 1960; Wang et al., 2007; Luo, 2012) where temperature (*x*_1_), methanol concentration (*x*_2_) and induction time (*x*_3_) were selected as the independent variables, while periplasmic specific ASNase II activity (U.g^−1^) and biomass concentration (g_dcw_.L^−1^) as the responses (Table 1). According to this design, each variable was varied at three levels, namely 15, 20, and 25°C for *x*_1_, 1, 2, and 3% (v/v) for *x*_2_, and 48, 72, and 96 h for *x*_3_, giving a total of 11 runs including two repetitions of the central point to estimate the pure error. The results were analyzed by means of Statistica software, version 10 (StatSoft, Tulsa, OK, USA).

**Table 1:** 3^3−1^ Fractional factorial design used to investigate *P. pastoris* cultures in shake flasks.

**Run**	**Methanol concentration (% v/v)**	**Temperature (^**°**^C)**	**Induction time (h)**	**Periplasmic ASNase II activity (U.g^**−1**^)**	**Biomass concentration (g_**dcw**_.L^**−1**^)**
1	1.0	15	48	7.9	17.0
2	1.0	20	96	12.8	21.0
3	1.0	25	72	5.4	15.7
4	2.0	15	96	9.3	26.3
5^a^	2.0	20	72	12.9	23.9
6^a^	2.0	20	72	13.1	23.2
7^a^	2.0	20	72	12.7	24.2
8	2.0	25	48	7.1	20.7
9	3.0	15	72	5.1	31.1
10	3.0	20	48	16.2	18.1
11	3.0	25	96	7.8	22.0

*Independent variables: methanol concentration, temperature, and induction time; Responses: periplasmic specific ASNase II activity and biomass concentration*.

a *Repetition of central point*.

Cultures were carried out with about 1 g_dcw_.L^−1^ initial cell concentration in BMGY. After glycerol depletion, cells were harvested by centrifugation at 3,320 *g* for 10 min at room temperature, and the supernatant was discarded. After washing with 100 mM phosphate buffer, pH 6.0, the cell pellet was suspended in 50 mL of BMMY. The induction was performed under the conditions set by the experimental design (Table 1).

### *Pichia pastoris* Cultivation in Bioreactor

The inoculum was prepared by growing a stock culture of recombinant *P. pastoris* from 1.0 mL of unfrozen cell suspension in a 250-mL Erlenmeyer flask containing 50 mL of BMGY medium, at 30°C and 250 rpm in shaker incubator for 22 h. After that, 5 mL of cell suspension were used to determine the dry cell weight (g_dcw_) by means of a moisture analyzer, model MOC63u unibloc (Shimadzu, Kyoto, Japan). The volume of cell suspension ensuring an initial biomass concentration of 1 g_dcw_.L^−1^ was centrifuged at 3,320 *g*, and the pellet resuspended in BSMm in a 3 L bioreactor, model Bioflo 115 (New Brunswick, Edison, NJ, USA).

Cultures were performed on BSMm plus 40 g.L^−1^ glycerol and 13 g.L^−1^ (NH_4_)_2_SO_4_. The bioreactor was operated at 30°C during cell growth and starvation phases, and pH was monitored and controlled automatically at 5.0 with NaOH. During the induction phase, temperature was reduced at 20°C, and medium pH was maintained at 6.0.

In general aspects, the experimental conditions proposed in sections Cultures of *Pichia pastoris* in Shake Flasks, 3^3−1^ Fractional Factorial Design for the Induction Phase in Shake Flasks, and *Pichia pastoris* Cultivation in Bioreactor were selected based on the work of Ferrara et al. (2006).

### Determination of Oxygen Mass Transfer Coefficient

To evaluate the influence of oxygen mass transfer coefficient (*k*_L_*a*) on cell growth and ethanol production, three different *k*_L_*a* values, selected on previous screening of conditions (results not shown), were tested in bioreactor, namely 84 h^−1^ (1.0 vvm, 500 rpm); 115 h^−1^ (1.0 vvm, 600 rpm); and 160 h^−1^ (1.0 vvm, 700 rpm), in batch mode until glycerol depletion.

*k*_L_*a* was determined according to the gassing out method (Wise, 1951) by bubbling nitrogen into water (2.0 L) contained in the bioreactor to remove the dissolved oxygen, until the polarographic probe reached zero. *k*_L_*a* values were estimated at 30°C as the slope of the straight line obtained plotting ln(1-*C*/*C*_*S*_) vs. time (h) for different aeration rates and agitation conditions, being *C* the dissolved oxygen concentration at time *t* and *C*_*S*_ that at saturation (mg.L^−1^).

### Two Stage Fermentation

The bioreactor was operated in batch mode for 20 h with an initial medium volume of 1.5 L, after which fed-batch culture was performed with glycerol as carbon source for further biomass growth. During 50% (w/v) glycerol fed-batch phase, the specific feeding flow rate was varied in the range 5.6–12.6 mL.L^−1^.h^−1^ plus PTM solution at 15 mL_PTM_.L${}_{\text{glycerolsolution}}^{-1}$ and (NH_4_)_2_SO_4_ at 0.325 g_(NH4)2SO4_.g${}_{\text{glycerol}}^{-1}$ for 12 h. During both phases, the pH was maintained at 5.0 by NaOH addition, temperature at 30°C, aeration at 1.0 vvm, and agitation at 700 rpm, corresponding to *k*_L_*a* = 160 h^−1^.

Glycerol volumetric consumption rate (*r*_glycerol_, g_glycerol_.L^−1^.h^−1^) was calculated from the bioreactor mass balance by the equation (Dietzsch et al., 2011; Körner, 2013):

(3) $$r_{glycerol}=\frac{\mu_{set}}{Y_{x/s}}\cdot X\cdot e^{\mu_{set}\cdot (t-t_{f})}$$

where Y_x/s_ is biomass yield (g_dcw_.g${}_{\text{glycerol}}^{-1}$), *X* biomass concentration (g_dcw_.L^−1^), *t* the time (h), *t*_*f*_ the time when starting the feed (h), and μ_*set*_ the specific growth rate set for the fed-batch phase (h^−1^) defined as in Equation (1).

To ensure an exponential feeding profile during the growth phase, the volumetric feeding rate (*F*_i_, L.h^−1^) after the time interval Δ*t* (h) was calculated as:

(4) $$F_{i}=\frac{V_{i}}{C_{\text{glycerol}}}r_{\text{glycerol},\text{i}}$$

where:

(5) $$V_{i}=V_{i-1}+F_{i-1}\cdot \Delta t$$

being *V*_i_ and *V*_i−1_ the volumes of medium in the bioreactor (L) after and before Δ*t, C*_glycerol_ the glycerol concentration in the feed solution (g.L^−1^), *F*_i−1_ the volumetric feeding rate of glycerol solution before Δ*t*, and *r*_glycerol, i_ the glycerol volumetric consumption rate after Δ*t* (g_glycerol_.L^−1^.h^−1^).

### Induction of L-Asparaginase Biosynthesis

After a 1–2 h starvation period following the above high cell density culture, methanol fed-batch phase was performed feeding a 100% methanol solution at specific flow rates in the range 3.75–5.25 mL.h^−1^ plus 15 mL_PTM_.L${}_{\text{methanol}}^{-1}$ to induce ASNase II production. Different feeding regimes were tested, namely methanol addition by pulses up to 0.5 to 3% (v/v) followed by dissolved oxygen spikes and pseudo-continuous methanol feeding. During the induction phase, temperature was maintained at 20°C, aeration at 1.0 vvm, and agitation at 700 rpm, corresponding to *k*_L_*a* = 160 h^−1^.

### Analytical Methods

#### Biomass Quantification

Biomass dry weight was quantified by measuring the optical density at 600 nm using a microplate reader, model Spectramax® plus 384 (Molecular Devices, Sunnyvale, CA, USA). The weight was measured again, and the dry weight was calculated as the difference between the final and initial values.

Cell concentration in the samples was then calculated according to the following standard curve of optical density at 600 nm (OD_600_) vs. dry cell weight concentration (*X*), expressed in g_dcw_.L^−1^:

(6) $${\text{OD}}_{600}\text{ = 1.8738 X -0.0211}$$

The standard curve was obtained using a series of dilutions of homogenous *P. pastoris* cell suspension cultivated in BMGY from unfrozen stock as previously described. Cells were centrifuged at 3,320 *g* for 25 min at room temperature, washed twice and resuspended in distillated water. Ten milliliter of this suspension were dried at 55°C in a 15-mL pre-weighted dry Falcon tube until constant weight.

#### Glycerol Quantification

Glycerol concentration was determined by the Triglycerides Liquiform assay kit (Labtest Diagnostica, Lagoa Santa, MG, Brazil). Centrifuged samples (10 μL) were mixed with 1.0 mL of triglycerides assay kit and incubated at 37°C for 10 min. The optical density was read with the above microplate reader at 505 nm using distilled water as a blank. To prepare a standard curve, dilute solutions of pure glycerol (99.5%, from Synth®, São Paulo, Brazil) in the defined BSMm medium were used.

#### Ethanol and Methanol Quantification

Ethanol and methanol concentrations were determined by a gas chromatograph, model 6890N (Agilent Technologies, Wilmington, DE, USA), equipped with a fused-silica capillary column Poraplot Q (10 m × 320 μm × 5 μm), and a split-splitless injector. The carrier gas was helium at a pressure of 60 kPa in the injection port. The detector temperature was maintained at 280°C. The gas flow rate was set at 0.8 mL.min^−1^, and *n*-propanol used as internal standard. Injections for desorption of standards from needle trap device were made by splitless mode for 3 min at injection port temperature of 280°C, then the needle trap device was removed from the injector, and the system was switched into the split mode. The column oven temperature was held at 40°C for 5 min, ramped to 150°C at the rate of 10°C.min^−1^ and held at the final temperature for 5 min.

#### ASNase Activity Measurement

ASNase II activity was measured in whole cell suspension (periplasmic activity) based on asparagine hydroxylaminolysis, i.e., conversion of asparagine into β-aspartohydroxamate and ammonia. Samples (1.0 mL) of fermentation cultures were centrifuged at 3,320 *g* at 4°C for 10 min, and cells were resuspended in 0.9 mL of 20 mM Tris-HCl buffer (pH 6.8), 0.2 mL of 100 mM asparagine, and 0.2 mL of 1.0 M hydroxylamine (pH 7.0) (Dunlop et al., 1978; de Castro Girão et al., 2016). After incubation at 37°C for 30 min, 0.5 mL of trichloroacetic acid (TCA)/FeCl_3_ reagent (50 g/L TCA and 100 g/L FeCl_3_ in 0.66 M HCl) were added, and cell suspension centrifuged at 7,000 *g* for 10 min. Supernatant optical density was read at 500 nm with the same equipment as above (Dunlop et al., 1980). Enzyme activity was measured by β-aspartohydroxamate formation through a calibration curve. One unit (U) of ASNase II activity was assumed to be the amount of enzyme able to release 1 μmol of β-aspartohydroxamate per minute.

#### Enzyme Identification by SDS-PAGE

Samples of culture medium were assayed by SDS-PAGE in 12% polyacryamide gel (1.5 mm) to check the extracellular enzyme production and the purity degree (Sudhir et al., 2014). Protein bands were stained with Coomassie Brilliant Blue R-250 dye. Molecular mass bands were compared with those of Precision Plus Protein^TM^ Standards (Bio RAD, Hercules, CA, USA).

#### Protein Quantification

Protein concentration in the culture medium was determined using the Bicinchoninic Acid (BCA) Protein Assay kit (Sigma-Aldrich, St. Louis, MO, USA). Briefly, the protein sample (20 μL) was added to a 96 well plate containing 200 μL of the prepared BCA working reagent solution and incubated for 30 min at 37°C. Then, the optical density was read at 562 nm. Total protein concentration was obtained by a standard curve of several dilutions of a 1,000 mg.mL^−1^ bovine serum albumin (BSA) stock solution.

## Results and Discussion

### ASNase Production in Shake Flasks on Complex Medium

Heterologous protein expression in shake flask cultures of *P. pastoris* is usually performed in complex BMGY medium, whose composition, however, is not optimized; therefore, modifications have been proposed (Stratton et al., 1998; Pichia Fermentation Process Guidelines, 2002; Ghosalkar et al., 2008; Li et al., 2013). Even though *P. pastoris* can grow in a large range of pH (from 3.0 to 7.0) (Ahmad et al., 2014), our previous results (not shown) demonstrated that ASNase II activity did not significantly vary in the pH range 5.0–6.0. For this reason, the initial pH of the culture medium was adjusted to pH 6.0 (Pichia Fermentation Process Guidelines, 2002; Ferrara et al., 2006) to avoid salt precipitation occurring at higher pH (Cos et al., 2006).

It is noteworthy that the experimental design shows how independent variables and interactions among them can influence the output responses. A lot of different factors are often taken into considerations such as culture medium components in combination with operating parameters (i.e., temperature, induction time, agitation intensity, and aeration rate, among others). However, in the presence of many factors, it is not advisable to adopt a complete design from the beginning, but it is rather preferable to start with a fractional design trying to identify the actually significant factors. Another advantage of using an experimental design is that it allows simultaneously studying more factors with fewer experiments compared with several parametric variations. Such a reduction of the set of experiments can be described mathematically as 3^*n*−*k*^ + 2, where *n* is the number of factors to be investigated at the different levels (low, medium, and high) and *k* is the number of steps to reduce the experimental design (Mandenius and Brundin, 2008). Some of the factors highlighted above can influence protein expression in *P. pastoris*, i.e., temperature, MeOH concentration, induction time, pH, and medium composition (Jafari et al., 2011). For this reason, we employed a 3^3−1^ fractional factorial design to investigate the influence of methanol concentration, induction time and temperature on both periplasmic specific enzyme activity and biomass concentration in shake flasks (Table 1). Such a design, which is very useful to evaluate in a few experiments the simultaneous influence of independent variables on responses, is often used in bioprocess optimization (Mandenius and Brundin, 2008).

To describe the behavior of the system, second-order polynomial models were used to correlate independent variables with responses according to the equation:

(7) $$Y\;=\;\beta_{0}\;+\sum \beta_{i}x_{i}\;+\;\sum \beta_{ii}x_{i}^{2}+\;\sum \beta_{ij}x_{i}x_{j}$$

where *Y* is the response, β_0_ is the interception coefficient, β_i_ are the linear terms, β_ii_ are the quadratic terms, β_ij_ are the interaction terms, and *x*_i_ and *x*_j_ are the coded levels of the independent variables.

**Figure 1:**
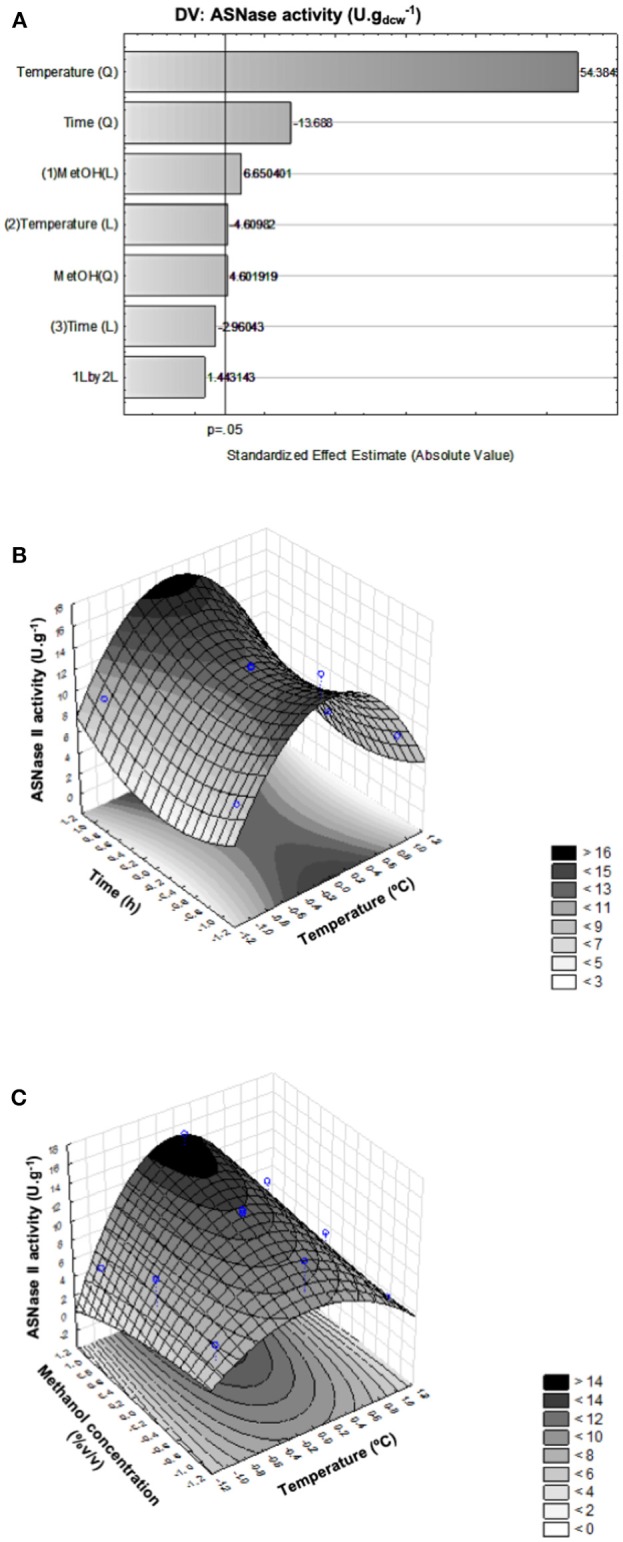
Pareto chart of ANOVA applied to periplasmic specific ASNase II activity (U.g^−1^) as response (Pure error = 0.037). Significant factors have a bar that spans beyond the vertical line (*p* > 0.05) **(A)**. Response surface plots of periplasmic specific ASNase II activity, showing the simultaneous influence of induction time (48, 72, and 96 h) and temperature (15, 20, and 25°C) **(B)**, and of methanol concentration (1, 2, and 3% v/v) and temperature (15, 20, and 25°C) **(C)** on this response.

Figure 1A, illustrates the Pareto chart of periplasmic specific ASNase II activity, in which the bar length is proportional to the standardized effect of the corresponding variable or interaction, and only bars beyond the vertical line correspond to statistically significant effects at 95% confidence level (*p* < 0.05). It can be seen that the positive quadratic effect of temperature was by far the strongest one on this response, followed by the negative quadratic effect of induction time, while all the others were not statistically significant or very weak.

It is possible to observe in the response surface plot of Figure 1C, that activity achieved maximum values at 20°C and gradually increased with methanol concentration. In contrast, Jafari et al. (2011) observed better anti-keratin 8 single-chain Fv TS1-218 production by *P. pastoris MUT*^*s*^ using methanol concentration below 3% (v/v) and lower temperatures (10–20°C).

On the other hand, consistently with the observations of the Pareto chart, albeit only a few significant, the induction time exerted a negative effect on the expression level, therefore induction times as shorter as possible (48 h) should be used to maximize it (Figure 1B). Therefore, taking into account the simultaneous influence of the above factors, we selected as the most favorable conditions for maximum ASNase II expression (16.2 U.g^−1^) within the selected ranges of independent variables a temperature of 20°C, a methanol concentration of 3% (v/v) and an induction time of 48 h.

### ASNase Production in Shake Flasks on Defined Medium

High cell density cultures of *P. pastoris* in bioreactor are usually carried out on chemically-defined high-salt concentration broth consisting of a modified basal salt medium (BSMm) enriched with a *Pichia* trace metal (PTM) solution (Ferrara et al., 2006; Julien, 2006; Ghosalkar et al., 2008; Li et al., 2013).

However, BSM has also some disadvantages, namely unbalanced composition, salt precipitation, and use of NH_4_OH as either nitrogen source or alkaline solution for pH control. NH_4_OH may be responsible for growth inhibition due to ammonia toxicity (Cos et al., 2006; Zhang et al., 2006; Ghosalkar et al., 2008; Santos et al., 2012). To face these constraints, NH_4_OH as nitrogen source was replaced by (NH_4_)_2_SO_4_ at concentration in the range 13–20 g.L^−1^, and the pH controlled by addition of NaOH (D'Anjou and Daugulis, 2000; Cos et al., 2006; Ghosalkar et al., 2008).

**Figure 2:**
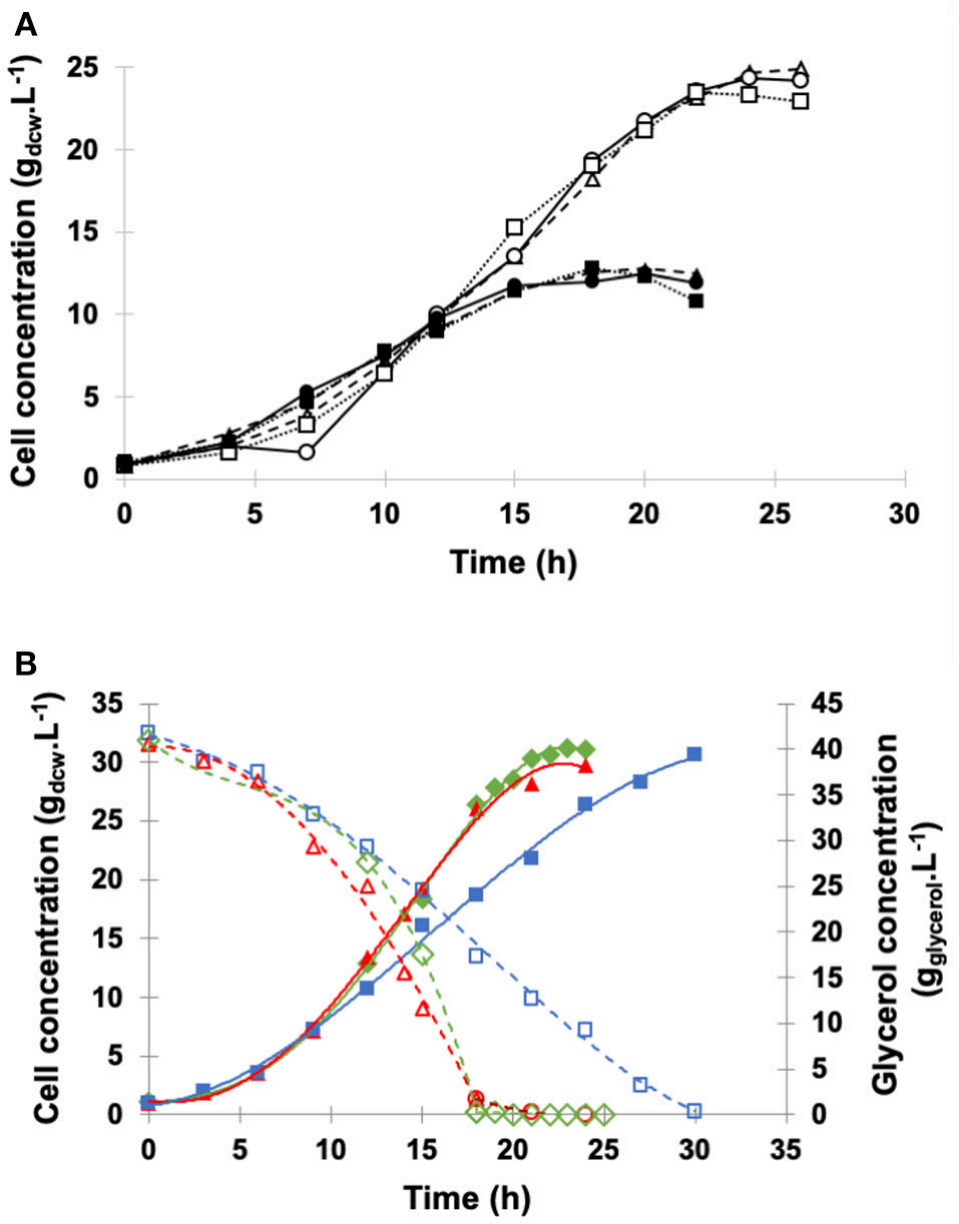
Growth curves of *P. pastoris*. **(A)** Shake flask cultures carried out at pH 5.0, 30°C, agitation of 250 rpm, inoculum concentration of 1.0 g.L^−1^ in: (Δ) BSMm with 13 g.L^−1^ (NH_4_)_2_SO_4_; (**°**) BSMm with 15 g.L^−1^ (NH_4_)_2_SO_4_; (□) BSMm with 20 g.L^−1^ (NH_4_)_2_SO_4_; (▴) SFMm with 13 g.L^−1^ (NH_4_)_2_SO_4_; (•) SFMm with 15 g.L^−1^ (NH_4_)_2_SO_4_; (■) SFMm with 20 g.L^−1^ (NH_4_)_2_SO_4_. **(B)** Bioreactor fermentations carried out on BSMm. Dry cell concentration: (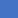) *k*_L_*a* = 84 h^−1^ (1.0 vvm, 500 rpm); (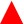) *k*_L_*a* = 110 h^−1^ (1.0 vvm, 600 rpm); (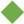) *k*_L_*a* = 160 h^−1^ (1.0 vvm, 700 rpm). Glycerol concentration: (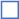) *k*_L_*a* = 84 h^−1^; (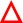) *k*_L_*a* = 110 h^−1^; (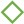) *k*_L_*a* = 160 h^−1^. Other conditions: temperature 30°C; inoculum concentration 1.0 g_dcw_.L^−1^; pH 5.0; broth volume 2.0 L.

Cell growth profiles in shake flasks using such BSMm and modified salt fermentation medium (SFMm) are shown in Figure 2A. In both culture media, *P. pastoris* grew similarly at the different NH${}_{4}^{+}$ concentrations, demonstrating the absence of any NH${}_{4}^{+}$ toxic effect within the tested concentration range. On the other hand, higher dry cell concentrations and cell yields were obtained in BSMm (24.1 ± 1.0 g_dcw_.L^−1^ and 0.60 ± 0.03 g_dcw_.g${}_{\text{glycerol}}^{-1}$) compared with SFMm (11.7 ± 0.9 g_dcw_.L^−1^ and 0.29 ± 0.02 g_dcw_.g${}_{\text{glycerol}}^{-1}$), while the pH decreased in both media from 5.0 to < 3.3. These values in BSMm medium were close to those reported in literature after glycerol depletion in BSM in bioreactor (Zhang et al., 2000b; Hélène et al., 2001; Ghosalkar et al., 2008). In BSMm the stationary growth phase was delayed (around 22–24 h) compared with SFMm (18 h) and lasted almost the same time as in defined medium (Hélène et al., 2001; Ferrara et al., 2006). The better growth of *P. pastoris* in BSMm is confirmed by a specific growth rate that was on average (0.153 h^−1^) about 7% higher than that obtained in SFMm (0.143 h^−1^). Based on these considerations, the better production results obtained in the former medium suggested us to select it for bioreactor experiments.

There was no ASNase II activity at the end of the induction phase because of strong acidity (pH ≤ 3.3) and low buffering capacity of both media. This result agrees with the observation that *S. cerevisiae* ASNase II periplasmic activity was fully inactivated at very low pH values (below 3.2) (Kim and Roon, 1983). As ASNase activity has an optimum pH of 7.0 (Dunlop and Roon, 1975; Ferrara et al., 2006), such acidic conditions were likely to alter its structure due to repulsion among positive charges, thereby distorting the catalytic site and reducing its activity. Since the lowest ammonium concentration (13 g.L^−1^) gave similar results to the others, suggesting no nitrogen limitation, it was selected for subsequent runs to decrease reagent costs.

### *Pichia pastoris* Cultivation in Bioreactor

To avoid a long oxygen limitation period in the bioreactor (Brierley et al., 1990; Chiruvolu et al., 1998), which would have led to ethanol accumulation (Brierley et al., 1990; Meagher and Inan, 2001; Looser et al., 2015) and consequent inhibition of recombinant protein induction, the growth phase on glycerol was divided in two stages, i.e., a glycerol batch phase (GBP) that allowed achieving a cell concentration >25 g_dcw_.L^−1^ and a glycerol fed-batch phase (GFP) during which glycerol was fed in limiting level and cell density was >50 g_dcw_.L^−1^. As a result, fermentations in bioreactor were carried out in three steps, namely GBP, GFP, and ASNase II production during methanol fed-batch phase (MFP), with only a short glycerol starvation period before starting MFP in BSMm.

#### Selection of Aeration Conditions

Taking into account that the aerobic growth of *P. pastoris* requires large amount of oxygen to ensure high cell density, to determine the best aeration and agitation conditions of GBP, three fermentations were carried out at 30°C in bioreactor on BSMm containing 13 g.L^−1^ (NH_4_)_2_SO_4_ and 40 g.L^−1^ glycerol under *k*_L_*a* of 84, 110, and 160 h^−1^ and controlling the pH at 5.0 to favor salt solubility (Brierley et al., 1990; Pichia Fermentation Process Guidelines, 2002; Ferrara et al., 2006). Figure 2B, shows the fermentation profiles until glycerol depletion.

Cell growth was very similar at *k*_L_*a* of 110 and 160 h^−1^, achieving similar biomass yields (Y_x/s_) (0.73 and 0.77 g.g${}_{\text{glycerol}}^{-1}$) and specific growth rates (μ) (0.19 and 0.21 h^−1^) (Table 2), while at the lowest *k*_L_*a* (84 h^−1^), despite the almost coincident Y_x/s_ values, μ and cell productivity were 42–48% and 24–28% lower, respectively, due to reduction of the oxygen level. These μ values fall in the typical range for *P. pastoris* grown on glycerol as the sole carbon source (Körner, 2013; Ahmad et al., 2014; Looser et al., 2015) and are close to that reported for *P. pastoris MUT*^*s*^ employed to produce ASNase II (Ferrara et al., 2006).

**Table 2:** *P. pastoris* biomass yield (Y_x/s_), specific growth rate (μ) and cell productivity in 3.0-L bioreactor containing 2.0-L BSMm plus PTM, at 30°C, pH 5.0, initial glycerol concentration of 40 g.L^−1^, and inoculum concentration of 1.0 g_dcw_.L^−1^.

***k*_**L**_*a* (h^**−1**^)**	**Y_**x/s**_ (g_**dcw**_.${\text{g}}_{\text{glycerol}}^{-1}$)**	**μ (h^**−1**^)**	**Cell productivity (g_**dcw**_.L^**−1**^.h^**−1**^)**
84	0.73	0.11	0.51
110	0.73	0.19	0.67
160	0.77	0.21	0.71

The yields obtained in bioreactor were appreciably higher when compared to those obtained in shake flasks on the same medium (results not shown).

#### Glycerol Feeding Profiles

To achieve high cell densities, while simultaneously avoiding ethanol formation, GFP was carried out based on GBP growth and biomass yields. According to D'Anjou and Daugulis (2000), ethanol accumulation can in fact be avoided when μ < $\frac{1}{2}$ μ_*max*_.

A glycerol volumetric consumption rate of 2.6 g_glycerol_.L^−1^.h^−1^ was calculated for our system by Equation (3) at *k*_L_*a* = 160 h^−1^, being μ_*set*_ = 0.072 h^−1^, and Y_x/s_ = 0.77 g.g${}_{\text{glycerol}}^{-1}$ under these conditions. To modulate substrate consumption, we assumed that the maintenance coefficient (*m*_s_) was < < μ during the exponential growth, μ and Y_x/s_ were constant, no oxygen limitation occurred during GFP, sampling volume, water evaporation and biomass dilution effect were negligible because of a volume variation <10% (D'Anjou and Daugulis, 2000).

To avoid excess ammonium inhibition (Zhang et al., 2000a; Cos et al., 2006), a 50% (w/v) glycerol solution containing 0.325 g_(NH4)2SO4_.g${}_{\text{glycerol}}^{-1}$ was fed to the bioreactor with an exponential feeding profile (Equation 4). Exponential feeding was simulated by a pseudo-exponential feeding with step increases every 2 h alternated to constant feed (13.1 mL.h^−1^). Using the μ and Y_x/s_ values from batch fermentations and assuming a dry cell concentration of 28 g_dcw_.L^−1^ and an initial volume of 1.5 L, the flow rate pattern summarized in Table 3 was experimentally tested.

**Table 3:** Values of the feed flow rate calculated by Equation (4) using μ = 0.072 h^−1^, Y_x/s_ = 0.77 g_dcw._g${}_{\text{glycerol}}^{-1}$, initial cell concentration of 28 g.L^−1^, and 50% (w/v) glycerol concentration, and tested in pseudo-exponential feeding runs.

**Time (h)**	**Flow rate (mL.h^**−1**^)**
0–2	8.4
2–4	9.9
4–6	11.6
6–8	13.6
8–10	16.0
10–12	18.9

#### High Cell Density Cultivations of *Pichia pastoris*

To perform high cell density cultures, the feed rate was set so as to ensure substrate-limited growth in GFP and subsequent *AOX1* promoter derepression in MFP (Tyagi et al., 2016). For this purpose, the same medium and conditions as in the batch runs were used including the same carbon-to-nitrogen ratio ensured by the selected feed solution.

**Figure 3:**
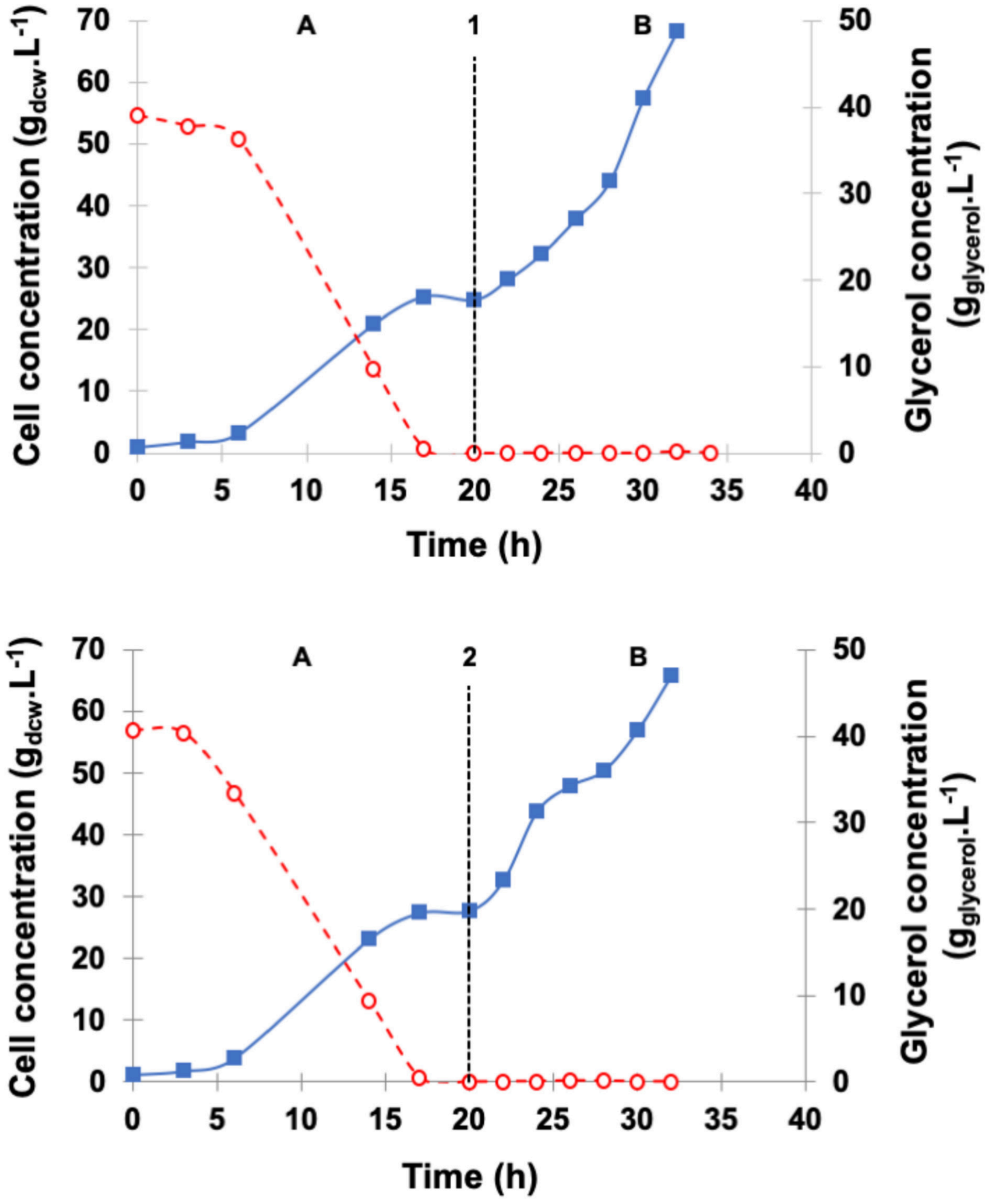
Dry cell concentration of *P. pastoris* (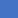) and glycerol concentration (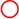) during high cell density cultures in bioreactor on BSMm. Conditions: temperature 30°C, inoculum concentration 0.82 g.L^−1^, pH 5.0, aeration 1.0 vvm, agitation 700 rpm (*k*_L_*a* = 160 h^−1^), and initial volume 1.5 L. **(A)** Glycerol batch phase with 40 g.L^−1^ glycerol for 20 h; **(B)** Glycerol fed-batch phase with 50% (w/v) glycerol solution with 0.325 g_(NH4)2SO4._g${}_{\text{glycerol}}^{-1}$ and 15 mL_PTM_.L${}_{\text{glycerolsolution}}^{-1}$ for 12 h. **(B1)** Glycerol fed-batch phase with pseudo-exponential feeding (average flow rate 13 mL.h^−1^), and **(B2)** Glycerol fed-batch phase at constant feed (13 mL.h^−1^).

Figure 3 illustrates the profiles of cell and glycerol concentrations in the medium throughout the fed-batch fermentation using the above described constant or pseudo-exponential pattern for a 12 h period. The GFP phase started after glycerol depletion (20 h) with both feeding strategies. During GFP, the values of specific growth rate (0.069 h^−1^ for the constant flow rate and 0.085 h^−1^ for the pseudo-exponential one) were substantially lower than that in the batch phase (0.219 h^−1^) just because glycerol was fed at growth-limiting level.

Cell concentration achieved 27.7 g_dcw_.L^−1^ at the start of constant feed and 65.8 g_dcw_.L^−1^ at the end of the 12 h period. On the other hand, the pseudo-exponential feed started after the batch period when cell density was 24.7 g_dcw_.L^−1^ and stopped after 12 h, reaching about the same cell concentration (68.3 g_dcw_.L^−1^) as the constant feed. At the end of fermentations, ethanol level was very low (< 0.2 g_ethanol_.L^−1^) and similar for the two feeding patterns.

### ASNase Production in Bioreactor

The objective of this part of the work was to induce ASNase II production by recombinant *P. pastoris* using methanol as the sole carbon source at high cell density. To ensure glycerol and ethanol depletion, the culture was submitted to a 1–2 h starvation period between GFP and induction phase. The same agitation speed and aeration rate as in the other phases (700 rpm and 1.0 vvm) were adopted, feeding pure methanol plus 12 mL_PTM_.L${}_{\text{methanol}}^{-1}$. Since our previous work demonstrated no significant difference in growth at pH 5.0 or 6.0 (results not shown), the latter value was selected to preserve the stability of produced enzyme. The culture was induced by means of methanol pulses (30 mL_methanol_.L${}_{\text{initialmedium}}^{-1}$) controlled by dissolved oxygen spikes (Stratton et al., 1998), which were performed after sudden dissolved oxygen increases.

**Figure 4:**
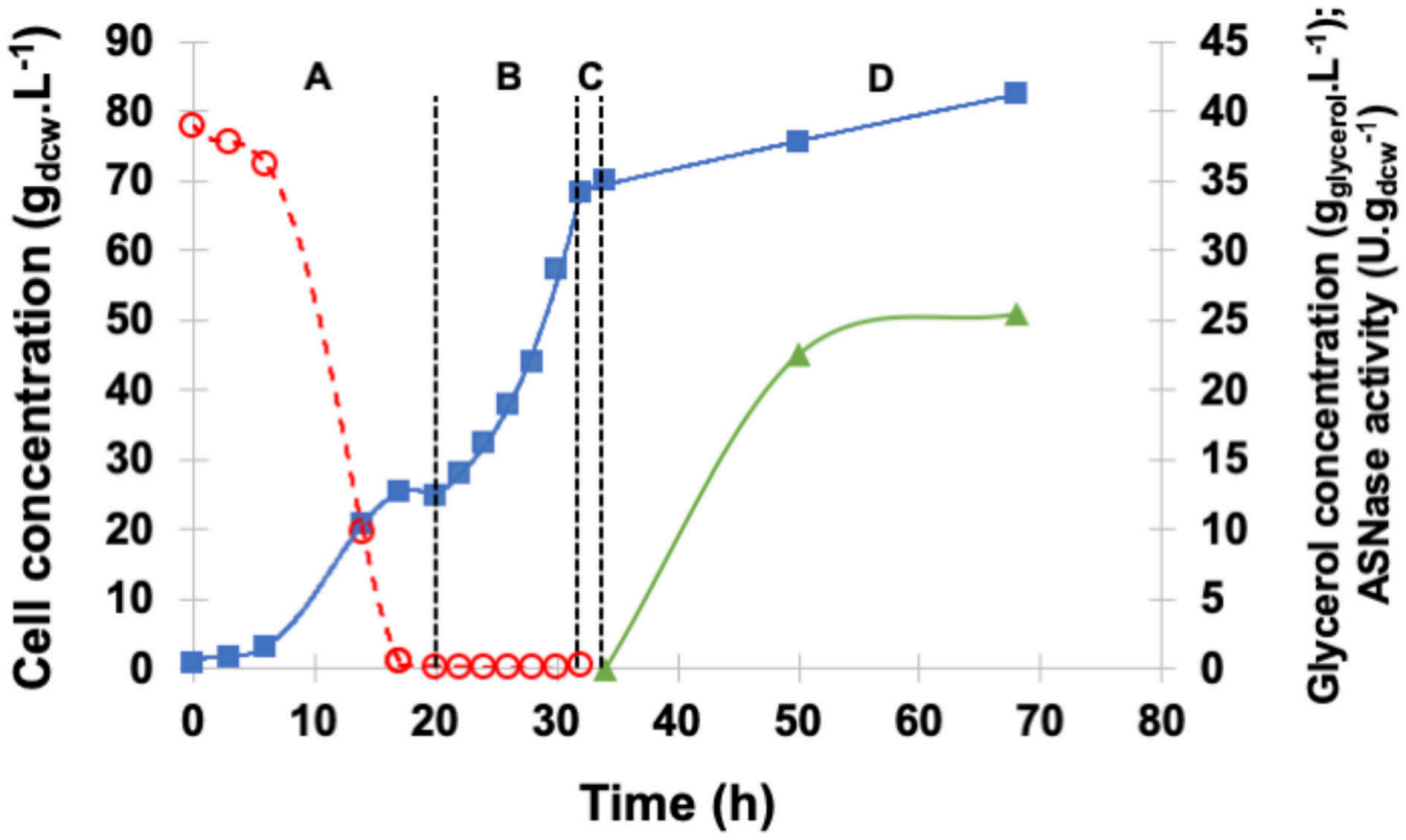
Kinetic profile of ASNase II production (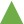) by recombinant *P. pastoris* in high cell density culture in bioreactor on BSMm under fed-batch methanol induction by pulses. Cell (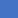) and glycerol (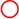) concentrations. **(A)** Glycerol batch phase with 40 g.L^−1^ glycerol level for 20 h; **(B)** Glycerol fed-batch phase at constant feed (13 mL.h^−1^) with 50% (w/v) glycerol solution with 0.325 g_(NH4)2SO4._g${}_{\text{glycerol}}^{-1}$ and 15 mL_PTM_.L${}_{\text{glycerolsolution}}^{-1}$ for 12 h; **(C)** Starvation period of 2 h. Conditions: temperature 30°C, inoculum concentration 0.82 g.L^−1^, pH 5.0, aeration 1.0 vvm, agitation 700 rpm (*k*_L_*a* = 160 h^−1^) and initial volume 1.5 L. (**D)** Induction fed-batch phase at 20°C, pH 6.0, with 100% methanol solution plus 12 mL_PTM_.L${}_{\text{methanol}}^{-1}$ fed by two pulses (30 mL.L^−1^) controlled by dissolved oxygen spikes.

The profiles of cell growth, periplasmic specific ASNase II activity, and glycerol concentration in the medium during fermentation are illustrated in Figure 4. GBP started with an initial glycerol concentration of 40 g.L^−1^ and lasted 20 h, GFP was performed with exponential feed rate for 12 h, and, after the starvation period, glycerol and ethanol were totally consumed, while cell concentration reached 70 g_dcw_.L^−1^.

After 34 h of fermentation, i.e., at the end of glycerol starvation period, the culture was induced for 34 h. The growth rate was low during the induction phase, at the end ASNase II specific activity (25.4 U.g${}_{\text{dcw}}^{-1}$) was more than twice that detected in shaker, and cell concentration was as high as 82.5 g_dcw_.L^−1^.

#### ASNase Induction by Methanol Pulses

The use of methanol pulses was reported to be a simple and effective strategy to produce recombinant proteins (Dietzsch et al., 2011). Therefore, it has been adopted in the production step performed at 20°C and pH 6.0, while keeping all the other growth conditions unvaried including constant flow rate (13.1 mL.L^−1^) in GFP.

**Figure 5:**
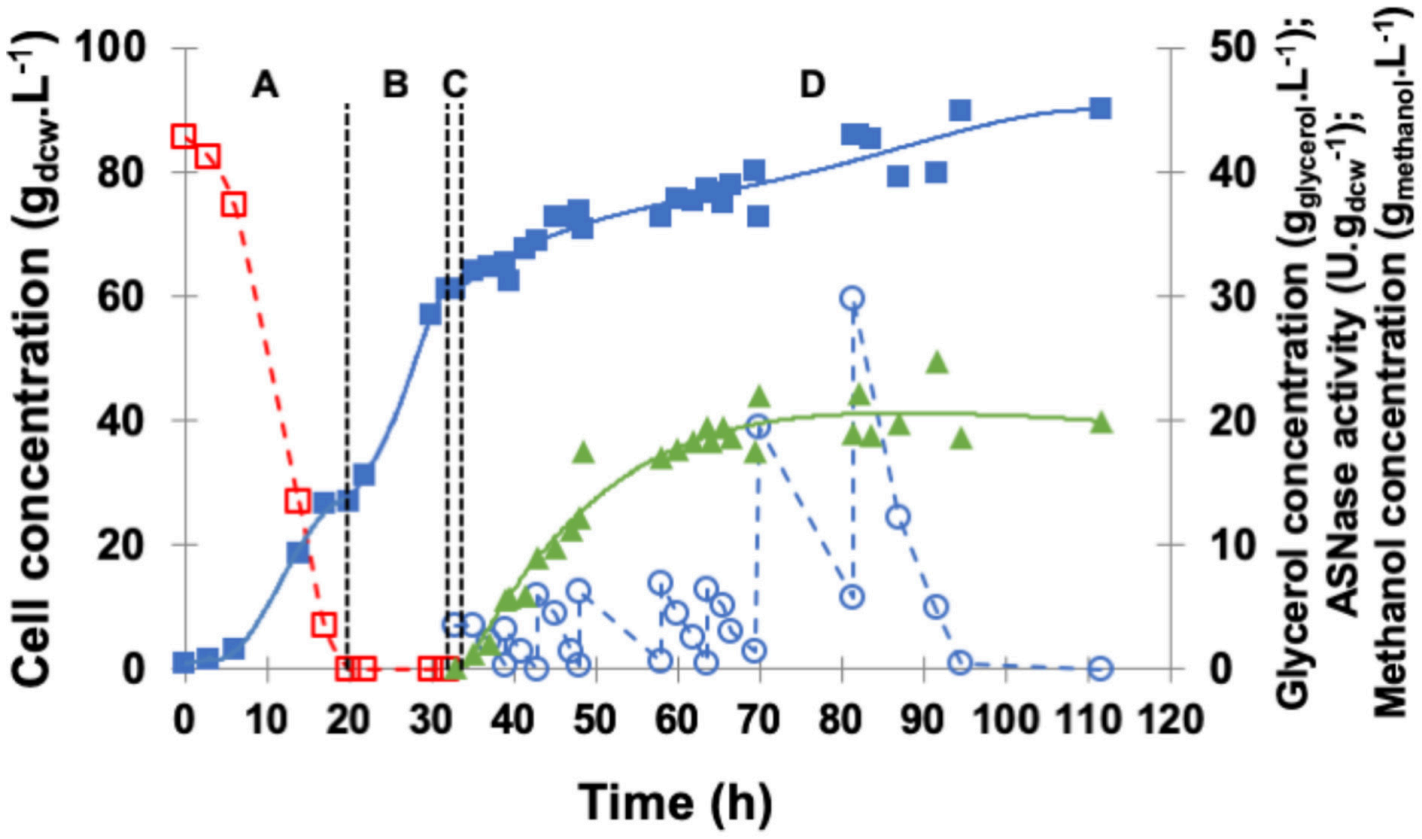
Kinetic profile of ASNase II production (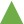) by recombinant *P. pastoris* in high cell density culture in bioreactor on BSMm under fed-batch methanol induction by pulses. Cell (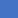) and glycerol (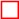) concentrations. **(A)** Glycerol batch phase with 40 g.L^−1^ initial glycerol level for 20 h; **(B)** Glycerol fed-batch phase at constant feed (13 mL.h^−1^) with 50% (w/v) glycerol solution with 0.325 g_(NH4)2SO4._g${}_{\text{glycerol}}^{-1}$ and 15 mL_PTM_.L${}_{\text{glycerolsolution}}^{-1}$ for 12 h; **(C)** Starvation period of 1 h. Conditions: temperature 30°C, inoculum concentration 0.91 g.L^−1^, pH 5.0, aeration 1.0 vvm, agitation 700 rpm (*k*_L_*a* = 160 h^−1^) and initial volume 1.5 L. **(D)** Induction fed-batch phase at 20°C, pH 6.0, with 100% methanol (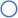) solution plus 12 mL_PTM_.L${}_{\text{methanol}}^{-1}$ fed by pulses: 2 small pulses (5 mL.L^−1^), 4 medium pulses (10 mL.L^−1^), and 2 large pulses (30 mL.L^−1^) controlled by dissolved oxygen spikes.

The profiles of cell, glycerol, and methanol concentrations as well as periplasmic specific ASNase II activity are illustrated in Figure 5. GFP started after glycerol depletion (20 h) when cell concentration was 27.0 g_dcw_.L^−1^. After the starvation period (1 h), glycerol and ethanol were absent, and cell concentration reached 61.0 g_dcw_.L^−1^ before the induction phase.

At the end of induction, specific ASNase II activity reached 24.1 U.g${}_{\text{dcw}}^{-1}$ and cell concentration 90.1 g_dcw_.L^−1^. After the first methanol pulse, a 2-h adaptation period was observed during which methanol was not consumed. Multiple pulses led to significant variations of μ that ranged from only 0.006 h^−1^ in the first pulse to 0.039 h^−1^ in the last one, with an average value throughout the whole induction phase of 0.033 h^−1^. After every methanol pulses, dry biomass concentration slightly decreased, and dissolved oxygen level dropped to < 10%, confirming methanol toxicity and the high oxygen requirement of methanol metabolism (Bushell et al., 2003). Nonetheless, ASNase II levels were higher than in shake flasks and close to those detected with only two methanol pulses (30 mL.L^−1^), which confirms the important role of oxygen in proteins induction and of high cell density in gene expression (Zhang et al., 2000b; Dietzsch et al., 2011). Ferrara et al. (2006) reported for ASNase II production in shake flasks glycerol depletion after 22–24 h, μ of 0.30 h^−1^ and Y_x/s_ of 0.56 g.g${}_{\text{glycerol}}^{-1}$. During cell growth, pH decreased from 6.0 to 2.9, and before induction by methanol addition it was adjusted to 6.0 with 1 M KOH. During the induction phase, a low cell growth rate and a gradual decrease of pH were observed. ASNase II levels were very high after 3 h of induction (394 U.g${}_{\text{dcw}}^{-1}$), reaching 562 U.g${}_{\text{dcw}}^{-1}$ in 5 h. Finally, using methanol pulses, a maximum specific growth rate of 0.013 h^−1^ was obtained under the tested conditions, with no methanol limiting concentrations.

**Figure 6:**
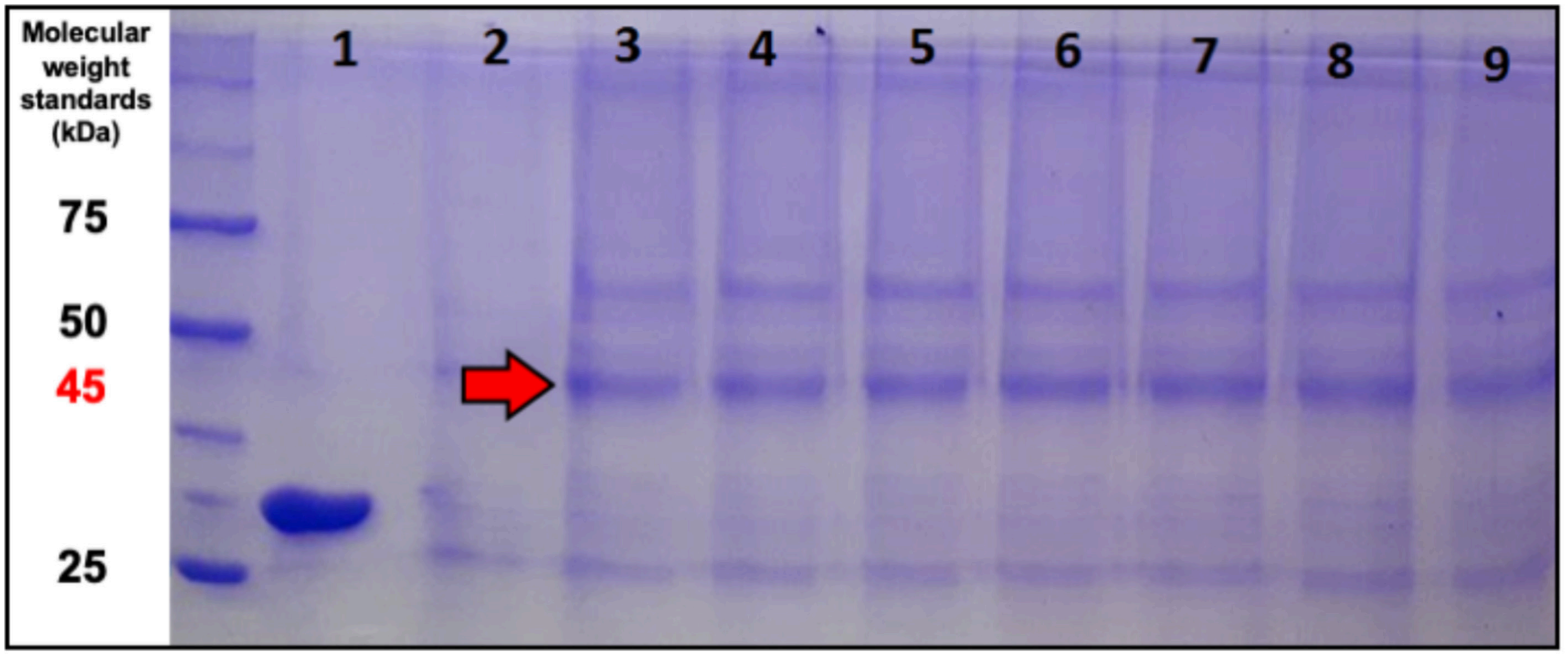
SDS-PAGE of supernatant samples stained with Coomassie Blue. **(1)** Commercial *E. coli* ASNase taken as a control; **(2)** supernatant sample just before induction. Supernatant samples taken just after induction by: **(3)** 1^st^ pulse; **(4)** 2^nd^ pulse; **(5)** 3^rd^ pulse; **(6)** 4^th^ pulse; **(7)** 5^th^ pulse; **(8)** 6^th^ pulse; and **(9)** 8^th^ pulse.

To evaluate ASNase II presence in the extracellular medium, SDS-PAGE of supernatant samples taken at the end of each pulse was performed. A band around 45 kDa was detected in all samples collected after the induction (red arrow in Figure 6). This value is only a little higher than the molecular weight (38.7 kDa) reported for *S. cerevisiae* ASNase II, a tetramer formed by four identical subunits each containing 362 amino acid residues (Dunlop et al., 1978; Ferrara et al., 2010), likely due to protein glycosylation (Ferrara et al., 2006; de Castro Girão et al., 2016). In fact, despite the lack of the original L-asparaginase signal sequence, the bioproduct was expected to be secreted or to accumulate in the periplasm. It is noteworthy that, since the extracellular enzyme activity was null (despite its presence), only the activity of the periplasmic enzyme was considered in this study. This interesting effect may be related to what was observed by Ferrara et al. (2006), who studied the expression of ASNase II in *S. cerevisiae*. These authors reported that non-secretion of protein (ASNase II) may have been due to an interaction of *S. cerevisiae* cell wall with some unidentified domain of ASNase II. Therefore, in the present case, it is possible that the enzyme secreted to the extracellular environment by *P. pastoris* may have undergone some modification to an inactive form. Likewise, Martínez et al. (2014) and Menéndez et al. (2013), who investigated the expression of exo-β-fructosidase from *Thermotoga maritima* by *P. pastoris*, observed secretion of this biomolecule into both the periplasmic space and the extracellular environment. On the other hand, there are some cases where proteins expressed by *P. pastoris* could not be secreted and remained in the periplasmic space, as it occurred for levansucrase (Lubineau et al., 1998) and human fucosyltransferase (Trujillo et al., 2001). According to the literature (Cereghino and Cregg, 2000; Cereghino et al., 2002), the efficiency of secretion depends not only on factors that direct the heterologous protein to the culture medium, but also on the nature of the protein structure.

#### ASNase Production Induced by Continuous Methanol Feeding

Methanol concentration is a critical parameter in *P. pastoris* cultivation since it influences both growth and heterologous gene expression under *AOX* promoter (Jahic et al., 2002; Cos et al., 2006). Some authors suggested to operate under conditions ensuring μ_max_ in methanol induction phase (Looser et al., 2015), while others reported that they are not necessary for high expression levels. On the other hand, the dissolved oxygen level in the broth seems to be widely accepted as control parameter for methanol feeding.

**Figure 7:**
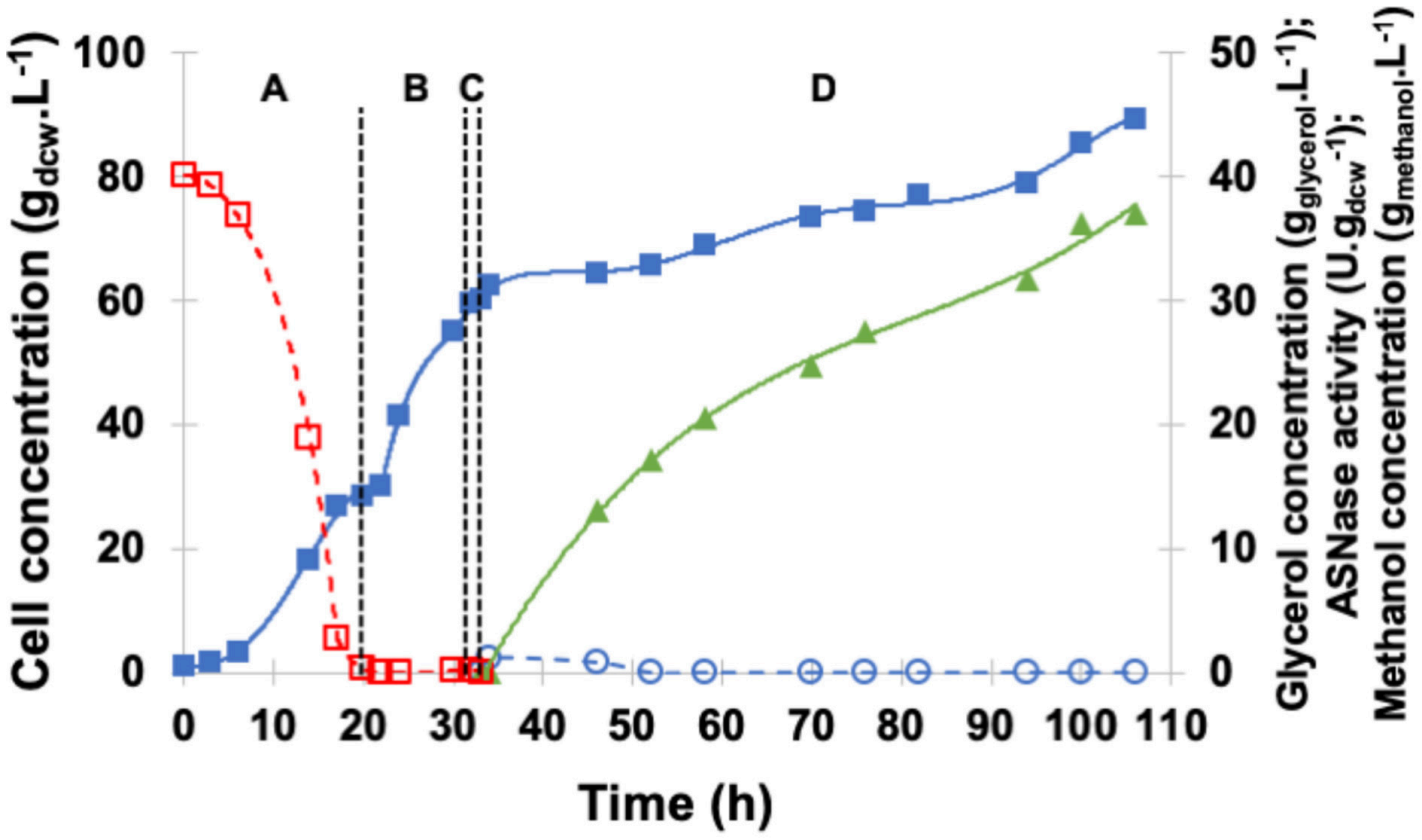
Kinetic profile of ASNase II production (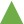) by recombinant *P. pastoris* in high cell density culture in bioreactor on BSMm under continuous methanol induction. Cell (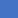) and glycerol (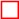) concentrations. **(A)** Glycerol batch phase with 40 g.L^−1^ initial glycerol level for 20 h. **(B)** Glycerol fed-batch phase at constant feed (13 mL.h^−1^) with 50% (w/v) glycerol solution with 0.325 g_(NH4)2SO4._g${}_{\text{glycerol}}^{-1}$ and 15 mL_PTM_.L${}_{\text{glycerolsolution}}^{-1}$ for 12 h; **(C)** Starvation period of 1 h. Conditions: temperature 30°C, inoculum concentration 0.95 g.L^−1^, pH 5.0, aeration 1.0 vvm, agitation 700 rpm (*k*_L_*a* = 160 h^−1^) and initial volume 1.5 L. **(D)** Induction fed-batch phase at 20°C, pH 6.0, with continuous feeding of 100% methanol (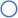) solution plus 12 mL_PTM_.L${}_{\text{mehtanol}}^{-1}$.

Figure 7 shows the profiles of cell, glycerol, and methanol concentrations as well as periplasmic specific ASNase II activity in the medium under induction by continuous methanol feeding. GFP was started after glycerol depletion (20 h) when cell concentration achieved 28.6 g_dcw_.L^−1^. After 1-h starvation and before induction, no glycerol or ethanol was detected, and biomass concentration reached 60.2 g_dcw_.L^−1^. For continuous induction at 20°C and pH 6.0, the feed rate was initially set at 2.6 mL.h^−1^ and then increased in such a way as to maintain dissolved oxygen level in the medium above 15% all the time (Zhang et al., 2000b; Bushell et al., 2003).

The average specific growth rate (0.005 h^−1^) under continuous methanol feeding was lower than that observed with pulses, cell concentration at the end of the 73-h induction period almost the same (89.4 g_dcw_.L^−1^), and specific ASNase II activity (37.1 U.g${}_{\text{dcw}}^{-1}$) 55% higher. These results confirm that μ_max_ is not necessarily an optimum condition for ASNase II production; oxygen availability seems, rather, to have played an important role in increasing ASNase II expression, by allowing cells to metabolize all methanol present in the medium avoiding the achievement of toxic levels (Tyagi et al., 2016). Moreover, continuous methanol feeding appeared to be the best strategy to ensure methanol-limited conditions, avoiding, at the same time, any oxygen limitation during MFP, and maximizing ASNase II expression.

Despite the lower specific ASNase II activity compared with *P. pastoris* carrying nitrogen de-repressed *S. cerevisiae ure2 dal80* (106 U.g${}_{\text{dcw}}^{-1}$), the ability of *P. pastoris MUT*^*s*^ strain used in this study to reach high cell density during fermentative growth allowed obtaining a volumetric activity (3,315 U.L^−1^) more than one order of magnitude higher than that (265 U.L^−1^) reported by Ferrara et al. (2004), and overall enzyme volumetric productivity of 31 U.L^−1^.h^−1^.

## Conclusions

*S. cerevisiae* L-asparaginase II was produced by *P. pastoris MUT*^*s*^ in multistage fed-batch cultivation divided in four distinct phases, namely glycerol batch, glycerol fed-batch, starvation, and methanol inducing phases. The main fermentation parameters determined in these phases were compared, namely specific growth rate, biomass yield, and enzyme activity. The recombinant *P. pastoris* strain used in this study showed optimum temperature for ASNase II production of 20°C, using 3% (v/v) methanol pulses every 24 h for a 48 h period in shake culture. In BSMm medium, biomass-to-glycerol yield was about 53% higher than in defined medium.

In glycerol batch fermentations under optimum *k*_L_*a* conditions, the specific growth rate was 0.21 h^−1^ and biomass yield 0.77 g.g${}_{\text{glycerol}}^{-1}$. Using these values for exponential growth, two feeding strategies, one based on an exponential feed profile and the other on a constant one, were adopted to ensure glycerol-limited conditions. Both were successful in achieving high cell densities (68.3 and 65.8 g_dcw_.L^−1^, respectively), although biomass yields (0.55 and 0.51 g.g${}_{\text{glycerol}}^{-1}$, respectively) were lower than in batch cultures. Using both glycerol feeding protocols ethanol concentration was negligible (< 0.2 g.L^−1^). The adoption of a starvation period before methanol feeding avoided any *AOX* repression due to the presence of ethanol or residual glycerol from glycerol fed-batch phase. The use of continuous methanol feeding for ASNase II induction allowed optimal control of methanol concentration in the medium, avoiding any oxygen limitation. Under these conditions, ASNase II was expressed at the highest levels. The results of this work demonstrate that not only methanol concentration but also oxygen availability in the induction phase are crucial for high recombinant protein production under the *AOX* promoter regulation.

## Author Contributions

DR, OP-P, KT-O, JF-S, and IS-M performed bioreactor and flask fermentations and analysis in BMGY, BSMm, and SFMm media, acquired the data, performed data calculations, standardization and assay of periplasmic specific ASNase II activity, interpreted the results and drafted the initial manuscript. MP and TB designed and constructed the recombinant *P. pastoris* system for ASNase expression. AC, AL, GM, LF, and AP conceived and designed the study, interpreted the results, and drafted the manuscript. All authors revised and approved the final manuscript.

### Conflict of Interest Statement

The authors declare that the research was conducted in the absence of any commercial or financial relationships that could be construed as a potential conflict of interest.
