# Supplementary material for: Fed-Batch Production of Saccharomyces cerevisiae L-Asparaginase II by Recombinant Pichia pastoris MUTs Strain
Source: Front Bioeng Biotechnol. 2019 Feb 8;7:16. doi: 10.3389/fbioe.2019.00016 (PMC6375902; doi:10.3389/fbioe.2019.00016)
Supplement: Supplementary file 1 [file Data_Sheet_1.docx]

**SUPPLEMENTARY INFORMATION**

**Fed-batch production of *Saccharomyces cerevisiae* L-Asparaginase II**

**by recombinant *Pichia pastoris MUT^s^* strain**

David Rodrigues^1,†^, Omar Pillaca-Pullo^2,†^, Karin Torres-Obreque^2^,

Juan Flores-Santos^2^, Ignacio Sánchez-Moguel^2^, Marcela V. Pimenta^2^,

Tajindar Basi^3^, Attilio Converti^4^, André M. Lopes^5^, Gisele Monteiro^2^,

Luís P. Fonseca^1^, and Adalberto Pessoa-Jr^2^

^1^Bioengineering Department of Instituto Superior Técnico, Institute of Bioengineering and Biosciences, Universidade de Lisboa, Lisbon, Portugal.

^2^Department of Pharmaceutical-Biochemical Technology, School of Pharmaceutical Sciences, University of São Paulo, Brazil.

^3^Department of Pharmacy, King’s College London, London, United Kingdom.

^4^Department of Civil, Chemical and Environmental Engineering, Genova, Italy.

^5^Faculty of Pharmaceutical Sciences, University of Campinas, Brazil.

^†^The authors contributed equally in this work.

***Corresponding authors:**

Dr. André M. Lopes

[amorenilopes@gmail.com](mailto:amorenilopes@gmail.com)

Faculty of Pharmaceutical Sciences

University of Campinas, Brazil.

Prof. Dr. Adalberto Pessoa-Jr

[pessoajr@usp.br](mailto:pessoajr@usp.br)

Department of Pharmaceutical-Biochemical Technology

School of Pharmaceutical Sciences

University of São Paulo, Brazil.

**Cloning ASP3 gene in *pPIC9K* vector**

In order to obtain the *S. cerevisiae* type II L-asparaginase coding sequence in the mature form (amino acids 26-362) linked with a C-terminal histidine tag (Table S1 - *ASP3* gene sequence obtained from SGD Project, the following link also gives access to this information: <https://www.yeastgenome.org/locus/S000004145/sequence> [01/17/2019]), we decided to use as template a construct made previously by our research group, in which *ASP3 26-362* was inserted in *BamH I* and *Xho I* restriction sites of pET22b vector, allowing fusion of the gene with C-terminal his-tag vector sequence. Specific primers were designed to amplify the *ASP3_26_362 + his tag* gene adding restriction sites to *Avr II* and *Not I* in order to facilitate cloning steps into *pPIC9K*.

**Table S1.** *S. cerevisiae* type II L-asparaginase coding sequence in the mature form (amino acids 26-362) linked with a C-terminal histidine tag (underlined sequence).

| **ASP3_26-362 + his DNA sequence**  **(1032 bp)** | GAAGAGAAGAATTCTTCTTTGCCATCAATCAAAATTTTTGGTACCGGCGGTACTATCGCT TCCAAGGGTTCGACAAGTGCAACAACGGCGGGTTATAGCGTGGGATTAACCGTAAATGAT TTAATAGAAGCCGTCCCATCTTTAGCTGAGAAGGCAAATCTGGACTATCTTCAAGTGTCT AACGTTGGTTCAAATTCTTTAAACTATACGCATCTGATCCCATTGTATCACGGTATCTCC GAGGCACTAGCCTCTGATGACTACGCTGGTGCGGTTGTCACTCATGGGACCGACACTATG GAGGAGACAGCTTTCTTCTTAGATTTGACCATAAATTCAGAGAAGCCAGTATGTATCGCA GGCGCTATGCGTCCAGCCACTGCCACGTCTGCTGATGGCCCAATGAATTTATATCAAGCA GTGTCTATTGCTGCTTCTGAGAAATCACTGGGTCGTGGCACGATGATCACTCTAAACGAT CGTATTGCCTCTGGGTTTTGGACAACGAAAATGAATGCCAACTCTTTAGATACATTCAGA GCGGATGAACAGGGATATTTAGGTTACTTTTCAAATGATGACGTGGAGTTTTACTACCCA CCAGTCAAGCCAAATGGATGGCAATTTTTTGACATTTCCAACCTCACAGACCCTTCGGAA ATTCCAGAAGTCATTATTCTGTACTCCTATCAAGGCTTGAATCCTGAGCTAATAGTAAAG GCCGTCAAGGACCTGGGCGCAAAAGGTATCGTGTTGGCGGGTTCTGGAGCTGGTTCCTGG ACTGCTACGGGTAGTATTGTAAACGAACAACTTTATGAAGAGTATGGTATACCAATTGTT CACAGCAGAAGAACAGCAGATGGTACAGTTCCTCCAGATGATGCCCCAGAGTACGCCATT GGATCTGGCTACCTAAACCCTCAAAAATCGCGTATTTTGCTACAATTATGTTTGTACTCC GGCTACGGCATGGATCAGATTAGGTCTGTTTTTTCTGGCGTCTACGGTGGTCACCACCAC CACCACCACTGA |
| --- | --- |
| **Asparaginase with histidine tag (343 amino acids)** | EEKNSSLPSIKIFGTGGTIASKGSTSATTAGYSVGLTVNDLIEAVPSLAEKANLDYLQVS NVGSNSLNYTHLIPLYHGISEALASDDYAGAVVTHGTDTMEETAFFLDLTINSEKPVCIA GAMRPATATSADGPMNLYQAVSIAASEKSLGRGTMITLNDRIASGFWTTKMNANSLDTFR ADEQGYLGYFSNDDVEFYYPPVKPNGWQFFDISNLTDPSEIPEVIILYSYQGLNPELIVK AVKDLGAKGIVLAGSGAGSWTATGSIVNEQLYEEYGIPIVHSRRTADGTVPPDDAPEYAI GSGYLNPQKSRILLQLCLYSGYGMDQIRSVFSGVYGGHHHHHH* |

The PCR product revealed a band of approximately 1032 bp in the agarose gel (Figure S1). After this step, we proceeded with the cloning of gene into the *pPIC9K* vector between the *Avr II* and *Not I* restriction sites.


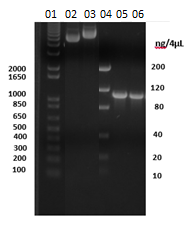


**Figure S1.** PCR product of the *ASP3_26-362 + his*. Lane 01: 1 kb plus DNA ladder marker. Lanes 02 and 03 *pPIC9K* plasmid miniprep; 04 Low Mass DNA ladder marker. Lane 05: PCR product of the *ASP3_26-362 + his* gene. The numbers at the left side of picture indicate the base pairs quantity.

The *ASP3_26-362 + his* sequence was cloned in-frame position with the α-mating Factor secretion signal (displayed in the vector diagram of Figure S2). The correct sequence insertion into *pPIC9K* was confirmed by DNA sequencing. Following, the recombinant *pPIC9K* + *ASP3_26-362* + his plasmid was linearized with *Sal I* enzyme and successfully transformed into *P. pastoris* KM71 to generate a HIS^+^ *MUT^S^* strain. As negative control, an empty *pPIC9K* was also linearized and transformed in the same *P. pastoris* strain.


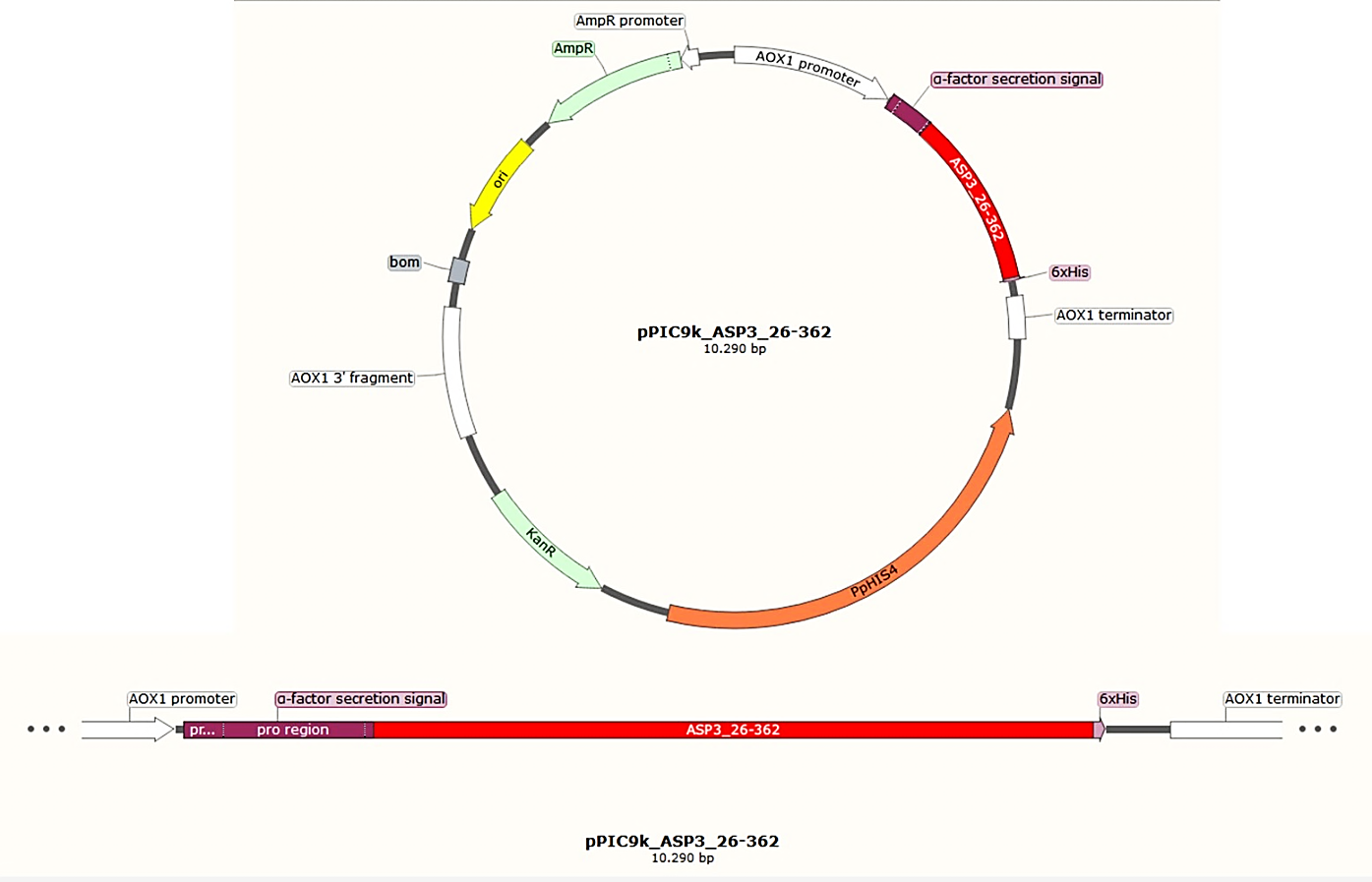


**Figure S2.** Vector diagram showing the *ASP3_26-362 + his* sequence in-frame with α-Factor secretion signal. PpHIS4: *P. pastoris* wild-type gene coding for histidinol dehydrogenase (~2.4 kb) present in *pPIC9K* vector used to complement *P. pastoris* his4 strains. KanR: Kanamycin resistance gene from Tn903 that confers resistance to geneticin in *P. pastoris*. AmpR: Ampicillin resistance gene to allow selection of bacteria transformants. This figure was made through the SnapGene^®^ software (from GSL Biotech, available at: snapgene.com).

Afterwards, 96 yeast transformants were screened to geneticin resistance. One clone exhibited resistance to 4 mg/mL geneticin. To confirm whether integration of the *pPIC9K* + *ASP3_26-362* + his was correctly inserted in the *P. pastoris* genome, the genomic DNA of this clone was used as template in a PCR reaction with the primers flanking the insertion site in the genome at the *AOX* locus. The amplified product revealed a band of approximately 1650 bp in agarose gel (Figure S3, lane 3), in contrast with 650 bp of the negative control. The difference of approximately 1000 bp found is due to the presence of *ASP3_26-362 + his* sequence and confirms the correct integration of the vector into the genome.


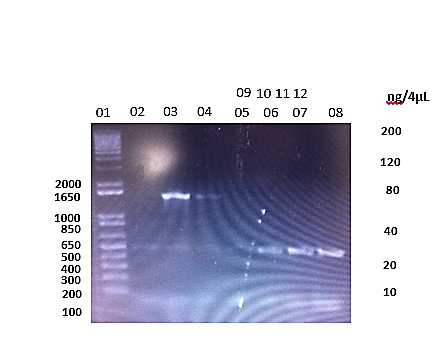


**Figure S3.** PCR product of *pPIC9K* + *ASP3_26-362 + his* genome insertion. Lane 01: 1 kb plus DNA ladder marker. Lane 03: genomic DNA from clone resistant to geneticin was used as PCR template. Lane 07: genomic DNA from *P. pastoris* transformed with *pPIC9K* empty vector used as OCR template. Lanes 02, and 04 to 06: genomic DNA from different yeast recombinant clones tested, not used in this work. The numbers at the left side of picture indicates the base pairs quantity.
